# Sex and gender differences in developmental programming of metabolism

**DOI:** 10.1016/j.molmet.2018.04.007

**Published:** 2018-04-30

**Authors:** Laura Dearden, Sebastien G. Bouret, Susan E. Ozanne

**Affiliations:** 1 University of Cambridge Metabolic Research Laboratories and MRC Metabolic Diseases Unit, Wellcome Trust-MRC Institute of Metabolic Science, Addenbrooke's Treatment Centre, Addenbrooke's Hospital, Level 4, Box 289, Cambridge, CB2 0QQ, United Kingdom; 2 The Saban Research Institute, Developmental Neuroscience Program & Diabetes and Obesity Program, Center for Endocrinology, Diabetes and Metabolism, Children's Hospital Los Angeles, University of Southern California, Los Angeles, CA, 90027, USA; 3 Inserm, Jean-Pierre Aubert Research Center, U1172, University Lille 2, Lille, 59045, France

**Keywords:** Pregnancy, Perinatal, Under nutrition, Obesity, Diabetes, Developmental programming, Sex differences

## Abstract

**Background:**

The early life environment experienced by an individual *in utero* and during the neonatal period is a major factor in shaping later life disease risk-including susceptibility to develop obesity, diabetes, and cardiovascular disease. The incidence of metabolic disease is different between males and females. How the early life environment may underlie these sex differences is an area of active investigation.

**Scope of review:**

The purpose of this review is to summarize our current understanding of how the early life environment influences metabolic disease risk in a sex specific manner. We also discuss the possible mechanisms responsible for mediating these sexually dimorphic effects and highlight the results of recent intervention studies in animal models.

**Major conclusions:**

Exposure to states of both under- and over-nutrition during early life predisposes both sexes to develop metabolic disease. Females seem particularly susceptible to develop increased adiposity and disrupted glucose homeostasis as a result of exposure to *in utero* undernutrition or high sugar environments, respectively. The male placenta is particularly vulnerable to damage by adverse nutritional states and this may underlie some of the metabolic phenotypes observed in adulthood. More studies investigating both sexes are needed to understand how changes to the early life environment impact differently on the long-term health of male and female individuals.

## 1 Introduction

### 1.1 Developmental programming of metabolic disease

Obesity and diabetes rates are increasing at an unprecedented rate around the world. While several genetic polymorphisms linked to obesity have been discovered [1], [2], these are few, only account for small increases in body weight, and explain less than 5% of the heritability of the condition. This suggests that environmental factors play a key role in determining risk of these conditions. The early life environment experienced by an individual *in utero* and during the neonatal period is now recognized as a major factor in shaping later life disease risk-including susceptibility to develop obesity, diabetes, and cardiovascular disease (see Table 1).

**Table 1:** Summary of reported sexually dimorphic phenotypes in humans (H), non-human primates (NHP), rodents (R), and sheep (S). The color of the box indicates whether the studies reported male (blue shading) or female (pink shading) to be more affected by the maternal environment.

An association between the early life environment and later life metabolic disease incidence was first reported by Hales and Barker, who proposed the “Thrifty Phenotype Hypothesis” based on their observations of an association between low birth weight (as a proxy for reduced fetal growth) and cardio-metabolic disease in adulthood [3], [4]. Further studies examining individuals who were *in utero* during the Dutch Hunger Winter (a famine in the Netherlands discussed in more detail later in this review) confirmed the association between *in utero* under-nutrition and the development of metabolic disease and suggested it was a causative relationship [5]. As well as the detrimental effects of exposure to under-nutrition *in utero*, there is now a wealth of evidence that demonstrates early life exposure to over nutrition - for example in cases of maternal obesity, diabetes or neonatal over nutrition - is also associated with increased metabolic disease incidence in individuals later in life. Comparative studies of siblings born before and after the mother underwent weight loss surgery have revealed that the children born after the mother had lost weight had greater insulin sensitivity, reduced adiposity, and reduced blood pressure compared to their siblings born when the mother was obese [6].

### 1.2 Sex differences in the incidence of obesity and diabetes

Globally, obesity is more prevalent in women than in men [7], although this varies by country, and, in the UK and USA, obesity is more prevalent in men [8], [9]. A recent study that tested 100 of the most common variants associated with differences in body size and shape found that 44 of the loci associated with waist to hip ratio - commonly associated with increased adiposity phenotypes - are differentially expressed in males and females [10], which may explain some of the variance in obesity rates. Sex differences in growth are apparent from the very early stages of development; male fetuses grow faster as early as the pre-implantation stage [11], demonstrating fundamental differences in growth and metabolism between the sexes. Some of this difference may be due to the genotype of an individual as the number of X chromosomes affects adiposity, suggesting that genes expressed on the X chromosome can regulate body weight [12], [13], [14]. Also, the female sex hormone estrogen has been widely suggested to be protective against obesity (discussed later). Furthermore, different sensitivities to metabolic hormones and adipokines such as leptin and insulin, which interestingly are often altered as a result of the nutritional environment in early life, may underlie some of the sex differences in prevalence of type 2 diabetes and obesity.

The purpose of this review is to summarize our current understanding of how the early life environment influences metabolic disease risk in a sex specific manner. We also discuss the possible mechanisms responsible for mediating these sexually dimorphic effects and highlight the results of recent intervention studies in animal models and how these can be translated to a human scenario.

## 2 Sexual dimorphism in systems regulating energy homeostasis

### 2.1 The central nervous system

Perhaps unsurprisingly, differences in brain structure are inherent and present from before birth. Among the most basic difference is the observation that male neonates have a larger total brain volume than females [15]. The female sex hormone estradiol has been suggested to regulate neurogenesis and cell migration, as well as cell death during early development of many brain regions, including the hypothalamus. Absence or increased expression of the sex hormones during the perinatal period therefore causes permanent changes to neuronal architecture [16], [17], [18]. Estrogen is produced initially by the corpus luteum and later by the placenta and is maintained at a high level throughout pregnancy such that both sexes are exposed equally. The primary source of the male sex hormone testosterone is the fetal Leydig cells, which develop in males just after sex determination; in humans, by 9 weeks post conception, genitalia have begun to develop and testosterone is produced [19]. A perinatal testosterone surge occurs in humans in the second trimester [20] and in rodents in the first week of neonatal life (which interestingly in both species coincides with the timing of development of hypothalamus [21], [22]). In males, during gestation, testosterone enters the brain and is converted to estradiol via aromatase. This process is responsible for masculinization of the brain [23]. Diet and sex specific factors continue to interact to regulate neurogenesis in the adult hypothalamus, when estradiol appears to have an inhibitory effect on neurogenesis [24], [25], [26].

The sex hormones potently control food intake and body weight [27]. In general, male animals are larger and consume more food than females, and even when expressed as kilocalories per gram body weight, daily food intake is greater in male than female rodents [28]. This is partly explained by the fact that estrogen exerts an inhibitory effect on meal size and daily food intake, as well as regulating diurnal feeding patterns [29], [30]. The hypothalamus, a brain region essential for the regulation of energy homeostasis, is highly sexually dimorphic. This dimorphism is not just restricted to the areas of the hypothalamus that are responsible for regulating fertility and reproduction. For instance, pro-opiomelanocortin (POMC) neurons within the arcuate nucleus of the hypothalamus (ARC) show differences in number and function between sexes, which may be in part due to the action of sex steroids on this neuronal population. Male mice have less anorexigenic POMC neurons within the ARC, which may explain their increased food intake compared to female animals. This seems to be an organizational effect of testosterone during development as neonatal testosterone administration to female mice results in a ‘masculinization’ and lowering of POMC neuron number within the ARC [31].

Gao and colleagues have shown that the number of excitatory glutamatergic synapses onto POMC neurons varies throughout the estrous cycle in mice, and this is likely due to changes in estrogen levels as injection of estradiol directly into the hypothalamus causes an increase in excitatory synapses onto POMC neurons [32]. Furthermore, Mackay et al. have shown that perinatal exposure to the estrogen-like compound BPA also causes rewiring within the ARC [33], similar to the well reported effects of leptin administration on the neonatal hypothalamus [34]. Glucose sensing in the hypothalamus is also different between the sexes, which is reported to be due to the effects that estrogen has on modulating glucose sensing at a cellular level [35], [36]. Differences in nutrient sensing and neuronal plasticity during development could result in different vulnerability to programming by the nutritional environment.

Recently, studies using genetically modified mice to investigate the role of different sub-populations of POMC neurons within the ARC in feeding control have revealed interesting sex differences in function. Shi et al. showed that leptin receptor expressing POMC neurons in females appear to be required for energy balance and fat distribution, whereas in male mice leptin receptor expressing POMC neurons are more important in maintaining glucose homeostasis [37], [38]. Furthermore, serotonin receptor expressing POMC neurons are responsible for regulating food intake, energy expenditure, and body weight in males, but only food intake in females [39]. Glutamatergic signaling in POMC neurons is thought to be a major way in which the activity of these neurons is altered in response to changing energy status, but knock out of the glutamate transporter VGlut2 on POMC neurons results in an increased body weight phenotype in male but not female mice [40], suggesting that glutamatergic signaling might not be as important in female animals. As POMC neuronal networks are both essential for regulating energy homeostasis and frequently shown to be regulated by the early life environment, sex differences in POMC neuron function warrant further investigation to see if they underlie sexually dimorphic phenotypes.

### 2.2 Metabolic hormones and adipokines

Circulating plasma leptin levels are different between males and females, but it is important to note that the pattern is the opposite way around in humans compared to rodents: in rodents, males have higher circulating leptin levels than females, whereas in humans, females have increased circulating levels of leptin for any degree of adiposity [27], [41], [42]. Females of both species are inherently more insulin resistant and have higher circulating insulin levels than males from birth [43], [44], [45], which explains why female rodents show a reduced anorectic response to intra-cerebroventricular insulin injection [46]. The phenotype of the neuronal insulin receptor knock-out mouse is different between male and female animals, with female animals showing hyperphagia and a greater increase in adiposity than male knock out animals [47]. Given the potential role of insulin as a programming factor, particularly in diabetic pregnancies [48], this could be extremely relevant to the sex specific prevalence of programming phenotypes.

### 2.3 Adipose tissue

Sex differences in the accrual of adipose tissue in different depots (for review see [49]) has significant effects on metabolic phenotype, as adipokine production, insulin sensitivity and free fatty acid (FFA) release varies between depots. The expression of leptin mRNA is increased in subcutaneous adipocytes compared to omental adipocytes, and the ratio of subcutaneous to omental leptin mRNA is higher in females than males [50]. Also, it has been shown that the metabolic rate per kilogram of adipose tissue is higher in women than men [51]. Some of the sex differences in adipose tissue are likely due to the sexually dimorphic role of adipocyte estrogen receptors in modulating adipose tissue expansion, inflammation, and fibrosis [52].

There are also differences in how the brain innervates peripheral organs such as adipose tissue, which may impact their function: pseudo-rabies neuronal tracing experiments show that males have more neurons projecting from the hypothalamus to abdominal fat, whereas females have more projections to subcutaneous fat [53]. Sex differences in adipose tissue are not limited to white adipose depots, as females have more brown adipose tissue and an enhanced capacity to beige their adipose tissue [54]. The mass changes that occur in adipose tissue gene expression in response to diet induced obesity are different between males and females, demonstrating significant differences in how obesity affects adipose tissue [55]. Given that the majority of programming phenotypes involves changes to the amount, distribution, or function of adipose tissue, sex differences such as these in the transcriptional response to a challenge such as obesity are an interesting potential underlying mechanism.

## 3 Sexually dimorphic responses to early life programming

### 3.1 Under nutrition and nutrient restriction during early life

Invaluable insight into the effects of exposure to under nutrition during the *in utero* period has been provided by studying individuals who were *in utero* during the Dutch Hunger Winter, which was a six-month famine that occurred in the western part of the Netherlands during the Second World War. The rapid onset and abrupt end of the famine has provided a unique opportunity to study the effects of under nutrition during specific time windows of pregnancy.

Individuals who were *in utero* during the famine display a range of disease phenotypes as adults, with many of the phenotypes varying depending on sex and the time of exposure to the famine (i.e. early or late gestation) [5]. Increased body mass index (BMI), waist circumference, and adiposity have been observed in females but not males who were exposed to the famine *in utero* [56], [57]. More recent studies have also shown a disrupted lipid profile (increased cholesterol and triglycerides) specifically in female individuals who were exposed to the famine [58].

Interestingly, there was an increased incidence of spina bifida and other central nervous system disorders in male neonates exposed to the famine [59], and as adults, famine-exposed males have been shown to have decreased brain volume [60], suggesting male vulnerability to neurological damage. This is similar to the significant amount of evidence that male babies born pre-term are more likely to suffer adverse neurological outcomes than females [61], [62]. In general, premature birth seems to result in more severe adverse outcomes in boys than girls [63], with increased mortality rates in male pre-term infants [61]. In surviving pre-term infants, body composition is altered independently of body weight, demonstrating that an increase in terms of gross weight of the baby is not sufficient to show recovery at the time of hospital discharge [64]. As decreased lean mass, which is most commonly seen in male pre-term infants, is associated with adverse neurological outcomes [65], this is an important issue to consider when treating and monitoring pre-term infants. Another study has reported increased skin fold thickness in pre-term males but not females at the equivalent of 36 weeks gestation [66], which may precede increases in adiposity through to adulthood.

Although many studies have been carried out in animal models on the effects of under nutrition or intra-uterine growth restriction (IUGR) on later life metabolic health, the results do not always corroborate the observations from human cohorts. Very early studies showed that among the offspring of pregnant rats underfed during early gestation, the males offspring displayed hyperphagia and obesity later in life, but female offspring were unaffected [67]. Maternal nutrient restriction in both rodents and sheep has also been reported to cause gender specific changes in gene expression in the liver and adipose tissue of fetuses, with males showing an increase in *11βhsd1*, *H6pd*, and *Cebpα* mRNA expression in response to nutrient restriction that is absent in female fetuses [68], [69]. In a non-human primate (NHP) model, maternal nutrient restriction near the end of pregnancy caused reduced growth of male but not female fetuses. Interestingly, the reduced fetal growth of male fetuses was associated with adipocyte hypertrophy and increased markers of white and brown cell adipogenesis [70]. Another recent paper studying an NHP model of IUGR showed that female offspring have increased total cholesterol, low-density lipoprotein, and subcutaneous fat. These changes were absent in male offspring, although they did display an increase in pericardial fat deposition [71]. In a study aiming to discover the effects of maternal under nutrition specifically during either gestation or the early post-natal period lactation, the authors reported that under nutrition during pregnancy caused increased adiposity and leptin levels in male and female offspring alike, whereas under nutrition restricted to the post-natal period had no effect on adiposity of male offspring but decreased adiposity of female offspring [72]. Further studies in an NHP model have shown male specific deficits in behavioral outcomes in response to maternal nutrient restriction during pregnancy [73], consistent with reports from the Dutch Hunger Winter studies that males are more vulnerable to adverse neurological outcomes.

### 3.2 Obesity and over nutrition during early life

Obesity is rapidly increasing among women of childbearing age [74], [75]; in many Western countries, one in five women is obese at the time of conception [76]. It has been known for some time that there is an association between maternal BMI and offspring BMI. High maternal BMI before and during pregnancy is a predictor of offspring obesity, adiposity, and metabolic syndrome as a young adolescent and as an adult [77], [78], [79]. Some authors have suggested that the link between maternal obesity and diabetes and offspring BMI is due simply to the effects of shared genetics or immediate effects on the child's birth weight. However, most recent studies find that adjustment for birth size does not attenuate associations between maternal obesity and childhood obesity. Furthermore, although some of the fetal–maternal association is due to shared genetics, a recent study has shown that genetic loci affecting the mother's weight independently affect offspring birth weight via the *in utero* environment independent of fetal genetics [80], [81].

A longitudinal analysis of children from obese pregnancies has reported increased body fat at 2–6 years of age in male children compared to children from a normal weight pregnancy. However, the adiposity of female children in the same study was not affected by maternal obesity [82]. Conversely, studies of maternal weight before pregnancy have demonstrated that the association between maternal pre-pregnancy BMI and offspring growth pattern from 0 to 7 years of age is stronger in females than males [83]. There is also considerable evidence that females are more susceptible to developmental programming by a diabetic mother or father [84], [85], [86], and this is discussed in more detail later.

The majority of animal models studying the effects of exposure to maternal obesity and/or over nutrition have reported results of mixed sex cohorts or focused exclusively on male offspring. There is now a wealth of evidence in animal models from rodents to NHP that maternal obesity causes increased adiposity, disrupted glucose homeostasis and cardiovascular dysfunction. Many rodent models of maternal (and even grandmaternal) obesity have suggested that females are more susceptible to programming of glucose homeostasis, whereas males are more susceptible to changes in adiposity and body weight [87], [88], [89]. However, one study that examined both sexes in a rodent model of maternal obesity reported that while both sexes are hyperphagic, female exposed animals showed a larger increase in food intake [90].

Many groups have reported that offspring exposed to maternal obesity show widespread changes in gene expression in the brain, and, interestingly, the few studies that have examined both sexes show that more genes are dysregulated in the hypothalamus (including *Crh* and *Glut3*) and forebrain in male than female offspring [89], [91]. Similarly, male animals exposed to neonatal over nutrition are more susceptible to develop hypothalamic inflammation [92]. The lack of inflammation in females may be in part due to estrogen, as this hormone is protective against free fatty acid-induced inflammation in primary neuronal cultures [92].

Although behavioral phenotypes have been less studied in the context of maternal obesity, Sullivan et al. have reported that maternal over nutrition results in increased anxiety like behavior in female offspring only in a NHP model [93]. The disparity between this observation and the previous studies that reported a stronger neuronal phenotype in male offspring of obese mothers may arise as a result of the apparent increased vulnerability of females to programming of the hypothalamic–pituitary–adrenal axis that is heavily involved in regulation of stress responses [94].

A recent study by Sun et al. revealed that male offspring exposure to a HFD during either gestation or lactation displayed decreased leptin sensitivity in the hypothalamus, whereas in female offspring, decreased leptin sensitivity was caused only by *in utero* exposure to HFD, revealing a sexual dimorphism in the timing of programming of leptin sensitivity that may be linked to sex differences in development [95]. A further study investigating the effects of exposure to maternal over nutrition during either gestation or the lactation period has shown female-specific programming of glucose homeostasis also occurs during the *in utero* period [89]. These studies highlight the importance of including both sexes in programming studies, as programming during the perinatal period clearly differentially affects offspring of each sex, and there may be different critical windows of development between sexes when energy homeostasis systems are particularly vulnerable to programming.

## 4 Mechanisms mediating sexual dimorphic programming

### 4.1 Epigenetics

Epigenetic regulation of the genome is integral to correct embryonic development. As some of the molecular machinery required for maintaining the epigenome is altered by nutritional status [96], [97], this represents a plausible mechanism by which changes in the *in utero* environment can be translated into permanent changes in gene expression in the developing offspring. During the pre-implantation stage of development, mass demethylation of the genome occurs in order to ensure the totipotency required for the rest of development. Interestingly, the timing of demethylation is different in the maternal and paternal pronucleus, with rapid demethylation of the paternal genome happening at an earlier developmental stage [98], [99]. This mass demethylation is followed by specific *de novo* methylation that remains relatively stable throughout the rest of life. The flexibility of epigenetic marks makes it possible for environmental factors to alter the epigenome in a sex specific manner, especially if the activity of the epigenetic machinery varies with nutritional state and by sex.

Differences in the expression levels of the DNA methyltransferase enzymes (DNMTs) are observed early in development between male and female blastocysts as well as at later developmental stages [100], [101], and these appear to be maintained through to adult life in at least some organs [102]. Differences in the activity of the DNMT enzymes may explain the dramatic sex differences in global genomic DNA methylation pattern that are evident in peripheral blood in adulthood [103]. Sex differences in epigenetic marks are not limited to those related to DNA methylation; widespread differences in histone modifications have been reported in the neonatal mouse brain [104]. The sex differences in DNMT levels may be due to the presence of sex hormones, as DNMT 1, 3a, and 3b are down regulated by progesterone [105]. A link between sex hormones and epigenetic machinery was confirmed recently in a study that showed intra-hippocampal infusion of estradiol increased DNMT3a and 3b expression, and also stimulated specific histone acetylation marks and altered the expression of Histone Deacetylase (HDAC) 1 and 2 [106].

Given how easily changes in the *in utero* environment could translate into changes in the epigenome, it is not surprising that many researchers have examined epigenetic modifications in the context of early life programming. Maternal nutrient restriction during pregnancy results in epigenetic modification of hepatic DNA and, consequently, gene expression [107], [108]. Maternal obesity causes an increase in expression of miRNA126 in adipose tissue that, in turn, leads to decreased translation of *Irs1* mRNA and therefore adipose tissue insulin resistance in these animals [109]. Sexually dimorphic differences in epigenetic determinants of chromatin structure in response to IUGR have been reported in the rodent cerebrum and hippocampus [110]. Furthermore, genome wide changes in methylation pattern have been observed in individuals who were *in utero* during the Dutch Hunger Winter, and the majority of these are sex specific, and could therefore explain the sexually dimorphic occurrence of obesity and other diseases in this cohort [111]. Further human studies have shown that maternal obesity alters the expression of miRNA210 in the placenta of female offspring specifically [112], [113]. Due to the widespread changes seen in global DNA methylation state as a result of changes in early life nutrition, several groups have examined the potential of methyl donor supplementation in the maternal diet during obese or nutrient-restricted pregnancies as an intervention strategy. Interestingly, these studies have reported sexually dimorphic effects on the growth of offspring [114], [115], as well as in DNA methylation levels in specific regions of the brain [115], [116].

In addition to the effects of maternal nutritional status at the time of conception and during pregnancy, there is emerging evidence that paternal nutritional state at the time of conception alters the sperm epigenome, thus providing a novel route through which paternal nutritional state can program changes in fetal gene expression [117], [118].

### 4.2 Placenta: a major source of sex differences

As the placenta develops early on in development from extra embryonic tissues, genetically it has the same sex as the fetus. This means that sex differences in the anatomy and growth of the placenta are apparent from the start of pregnancy; male placentas are physically smaller but more efficient when assessed by the placental weight required to support a fetus [119]. This may mean that male placentas have less reserve capacity in the face of a change in environment. Indeed, changes in placental morphology and increased placental inflammation in the offspring of obese mothers are stronger in male than female rodent offspring [120], and although the human male placenta ordinarily has a higher antioxidant capacity this is lost when exposed to maternal obesity [121]. A decreased placental size was reported among all individuals exposed to the Dutch hunger winter, but the reduction in size was greater in males than females [122], [123].

It has also been suggested that male placentas may be more susceptible to environmental changes because they have lower levels of the X-linked gene O- GlcNAc transferase (OGT), which is required in the placenta for some epigenetic processes. Reduced OGT expression could result in male placentas having less of the histone repressive mark H3K2me3, meaning that they are more susceptible to environmental changes that influence levels of this histone modification thus having greater consequences for placental function and development of the male fetus [124], [125]. However, not all studies have found the male placenta to be more susceptible to programming by the *in utero* environment. A recent study comparing placentas from lean and overweight women found increased placental weight and thickness, plus lower fetal-to-placenta weight ratio in overweight pregnancies, but these changes were seen only in female placenta [126]. Given the essential role that the placenta plays in nutrient transfer to the fetus and in supporting fetal growth, it is imperative that more research is conducted to understand how changes to nutritional state during pregnancy impacts differently in male and female placentae.

### 4.3 Role of metabolic hormones in developmental programming and how they could contribute to sex differences

Both circulating levels and the sensitivity to metabolic hormones varies vastly between sexes [46]. As these metabolic hormones also play integral roles in the development of major hypothalamic pathways required for maintaining energy homeostasis [127] and in responses to changing nutrient status (e.g. glucose levels), they have the potential to mediate some of the sex differences in phenotypes seen in response to changes in nutrition during the early life environment.

Rodents undergo a postnatal surge in circulating leptin levels that is independent of food intake but required for correct hypothalamic development [34]. In humans, leptin levels rise throughout gestation and peak just before birth. Interestingly, cord blood leptin levels are different between neonates as female babies have higher levels [128]. In mice, disruption of the post-natal leptin surge by neonatal leptin antagonism on post-natal day 9 causes sexually dimorphic effects on hypothalamic neuropeptide expression. Female animals show increases in *Bdnf*, *Cart*, and *LepR* mRNA as well as increased cell death in response to neonatal leptin antagonism on this specific post-natal day, but these effects are absent from male animals [129]. Blockage of the neonatal leptin surge by prolonged administration of a leptin antagonist also causes sexually dimorphic effects on hypothalamic neuropeptide expression [130]. At the other end of the scale, experimental hyperleptinemia induced during the neonatal period causes differential changes in renal function and thus blood pressure regulation in male and female mice but with a more severe hypertensive phenotype seen in female than male animals [131]. Although increased sympathetic tone appears to be responsible for the hypertension and subsequent renal interstitial damage in both sexes, renal sympathetic nerve denervation prevents interstitial damage in the kidneys in female mice only [131].

A recent paper by Samuelsson et al. suggests that melanocortin-4 receptor (MC4R) neurons in the paraventricular nucleus of the hypothalamus (PVH) are responsible for mediating the hypertensive phenotype often reported in the offspring of obese mothers [132]. This study also revealed interesting sex differences in the involvement of the melanocortin system in hypertensive phenotypes. Male MC4R knock-out mice showed an increased mean arterial pressure, whereas female MC4R knock-out mice did not. Furthermore, replacing MC4Rs specifically in the PVH resulted in an increased heart rate (HR) in female mice compared to full body MC4R KO animals, but this effect on heart rate was absent in male mice with a PVH specific MC4R reactivation. These results suggest that melanocortin signaling in the PVH mediates different aspects of cardiovascular function between the sexes and may explain why modification of signaling through this system by the early life environment causes different cardiovascular phenotypes in male and female offspring.

### 4.4 Protective actions of estrogens and how they may impact on susceptibility to early life programming

The estrogen family and its two respective receptors, ERα and ERβ, have been widely suggested to be protective against obesity, T2DM, and cardiovascular disease. This has been extensively reviewed elsewhere [133]. Estrogens have significant effects on insulin and leptin sensitivity and on the body's response to changes in glucose levels [46], [134], [135], [136]. As these metabolic hormones and nutrients are all implicated as ‘programming factors’ mediating the effects of sub-optimal nutrition during early life on long term health outcomes, the actions of estrogens in different tissues involved in maintaining energy homeostasis is a likely cause of the sexual dimorphism observed in susceptibility to early life programming.

One important cellular component that estrogens can exert significant effects on is mitochondria. Differences in mitochondrial number and function have been suggested to underlie the differences in life span between the sexes [137] and may also be responsible for some of the differences in response to the early life nutritional environment. Females have increased mitochondrial number in skeletal muscle, adipose tissue, and heart [138], [139], [140], and recent experiments have shown that the sex hormones have opposing effects on mitochondrial biogenesis in adipocytes where estradiol promotes and testosterone reduces mitochondrial proliferation [139]. Estradiol also directly modulates mitochondrial bioenergetic function and stimulates mitochondrial biogenesis in skeletal muscle [138], [141]. Overexpression of the estrogen receptor protects pancreatic β-cells from apoptosis by preserving mitochondrial function and suppressing endoplasmic reticulum stress in the face of cellular stress [142]. Despite this, maternal obesity appears to impact mitochondrial function in female offspring tissues more than in males. Female offspring of obese mothers are more susceptible to mitochondrial damage in their placenta, kidney, and skeletal muscle [112], [143], [144]. The source of mitochondrial damage in the offspring may be by transmission from the mother, as mitochondrial dysfunction has been reported in oocytes of obese mothers [144], [145].

Estradiol has been shown to have a neuroprotective role as it can regulate processes such as re-myelination and inflammation after brain injury [146], [147], [148]. This could mean that estradiol also has a protective role against some of the neuronal alterations caused by malnutrition in the early life period [149] and explains the high degree of sexual dimorphism seen in neuronal phenotypes, particularly in response to undernutrition during the early life period as discussed earlier. It has recently been shown that prenatal estradiol exposure induces hypothalamic insulin resistance in males [150]. Given that the high insulin levels in an obese pregnancy have been suggested as a strong candidate for programming alterations to hypothalamic pathways [48], an insulin desensitizing effect of estradiol would have significant effects on how maternal obesity impacts hypothalamic function differentially in males and females.

## 5 Are females more sensitive to changes in glucose availability during early life than males?

As more studies begin to investigate the effects of maternal over nutrition in both sexes, there is intriguing evidence emerging that females may be more sensitive to exposure to increased glucose levels during early life. Human studies have shown that females are more susceptible to programming by a diabetic mother or father than males [84], [85]. Furthermore, in a study investigating how maternal glucose tolerance is associated with offspring adiposity, increased adiposity was observed in male offspring of gestational diabetic mothers, but there was no increase in adiposity in male offspring if the mother only had “intermediate” glucose intolerance. However, female children show increased adiposity in response to “intermediate” maternal glucose intolerance, suggesting they may be more sensitive to increased, *in utero* glucose levels [86]. Given how many cases of gestational diabetes are thought go undiagnosed [151] and therefore unmanaged, this has important implications for the future health of babies, especially females.

Rodent studies have shown that a maternal diet high in sucrose during pregnancy has greater effects on metabolic phenotype in female offspring than males, causing widespread dysfunction to glucose homeostasis, adiposity and cardiac health [89], [152]. Similarly, a maternal high fructose diet caused decreased placental weight, along with increased plasma leptin, fructose and glucose levels in female offspring, but these phenotypes were not observed in male siblings [153].

The way that males and females handle changes to glucose levels as adults is very different. It is well known that counter regulatory responses to changing glucose levels are highly sexually dimorphic. In humans it has been shown that this is not due to different glycemic thresholds for hypoglycaemia detection, and could therefore be due to decreased inputs to the CNS from peripheral tissues such as adipose tissue and liver in females [154]. Furthermore, a recent study on the effects of diet on lifespan in Drosophila has shown that a high sucrose diet has a greater effect on shortening lifespan in females than males [155]. Therefore, males and females likely respond differently to glucose levels outside of the normal range when *in utero*, and this could explain the more severe phenotypes reported in female individuals across a range of species.

## 6 Sexual dimorphisms in intervention studies

Although more and more studies are emerging in the field of developmental programming trialling translatable interventions, few of these have reported the results of the intervention in offspring of both sexes. Several studies have shown that maternal exercise during an obese pregnancy can prevent some of the detrimental phenotypes in offspring, including a rescue of placental lipid deposition, and offspring glucose and insulin tolerance [156], [157]. So far, few groups have reported the effects of this intervention in both male and female offspring. The current studies suggest that maternal exercise has equally beneficial effects in both sexes on improving glucose tolerance [157], [158], but results in a rescue of hyperleptinemia and increased adiposity in male offspring only [159].

Administration of resveratrol to mothers during pregnancy has been shown to have positive effects on the placenta in an NHP model of maternal obesity [160], [161] and in preventing offspring obesity, glucose intolerance, and hypertension [162], [163]. To date, only one study has examined the effects of resveratrol supplementation during an obese pregnancy on both sexes, and have shown that resveratrol rescues increased body weight phenotypes in both sexes, but may affect different adipose depots [164]. A study examining the administration of resveratrol to mothers in a model of maternal protein restriction has reported positive outcomes in both sexes, but particularly in male offspring, in which resveratrol supplementation rescues the decreased body weight caused by maternal nutrient restriction [165]. However, some studies have urged caution over the administration of resveratrol during pregnancy, as it may have some negative outcomes if given to lean mothers [164] and on specific organ systems in the offspring of obese mothers [161]. Similar caution may be warranted over the use of metformin, which is commonly prescribed for gestational diabetes as it improves maternal outcomes such as insulin sensitivity and reduces gestational weight gain. Metformin is also being trialled as an intervention in obese pregnancies [166]. However, a recent study has shown that metformin prescribed for use during pregnancies complicated by polycystic ovary syndrome causes increased BMI in children at 4 years of age [167], showing that the use of metformin during pregnancy needs to also be carefully considered.

## 7 Conclusion

Pregnancies in which the nutritional state of the mother and fetus are compromised, be it over- or undernutrition, result in widespread detrimental effects to offspring long term cardio-metabolic health, and many of these effects are sexually dimorphic. Sex differences in the action of sex and metabolic hormones, the epigenome, and in how the placenta responds to a change in nutritional state during pregnancy are all likely candidates underpinning sexually dimorphic offspring phenotypes. Future research will elucidate whether the offspring phenotypes observed after interventions during a nutritionally compromised pregnancy are also sexually dimorphic. As in the field of metabolism as a whole, more studies investigating both sexes are urgently needed if we are to understand how changes to the early life environment impact differently on the long-term health of male and female individuals.
